# To Make Yourself Unhappy

**Published:** 1885-12

**Authors:** 


### HOW TO MAKE YOURSELF UNHAPPY.

In the first place, if you want to make yourself miserable, be selfish. Think all the time of yourself and your things. Don't care about anything else. Have no feelings for any but yourself. Never think of enjoying the satisfaction of seeing others happy, but rather, if you see a smiling face, be jealous lest another should enjoy what you have not. Envy every one who is better off than yourself; think unkindly toward them, and speak lightly of them. Be constantly afraid lest some one should encroach on your rights; be watchful apainst it, and if any should come near your things snap at them like a mad dog. Contend earnestly for everything that is your own, that may not be worth a pin Never yield a point. Be very sensitive, and take everything that is said to you in playfulness in the most serious manner. Be jealous of your friends lest they should not think enough of you; and if at any time they should seem to neglect you, put the worst construction upon their conduct.

